# Resolving the Interference of Anti‐CD38 Antibodies on Blood Compatibility Assays Using CD38 “Baitbodies” Approach

**DOI:** 10.1155/jimr/7343647

**Published:** 2026-01-04

**Authors:** Dianne Celine Gnann, Stephan Steinke, Hendrikus S. P. Garritsen, Michael Hust

**Affiliations:** ^1^ Abteilung Medizinische Biotechnologie, Institut für Biochemie, Biotechnologie und Bioinformatik, Technische Universität Braunschweig, Braunschweig, 38106, Germany, tu-braunschweig.de; ^2^ Institute for Clinical Transfusion Medicine, Celler Städtische Klinikum Braunschweig gGmbH, Braunschweig, 38114, Germany; ^3^ Fraunhofer Institute for Surface Engineering and Thin Films IST, Braunschweig, 38108, Germany

## Abstract

Blood transfusion safety depends on swift blood compatibility testing. Alloantibodies recognizing these blood group antigens determine blood compatibility, and the transfusion of incompatible blood can lead to life‐threatening hemolytic transfusion reactions. Recently, the landscape of blood compatibility testing has been complicated due to the interference caused by therapeutic antibodies targeting CD38, a key target in cancer immunotherapy that is also expressed on red blood cells. The presence of anti‐CD38 antibodies in blood samples has been found to bind to test or donor red blood cells, resulting in false positive tests. This interference poses a serious risk as it can potentially mask immunogenic alloantibodies and cause delays in supplying safe blood products. We present the development and application of fragment crystallizable (Fc)‐fusion constructs, referred to as “baitbodies”, designed to neutralize anti‐CD38 antibodies and mitigate false positive test outcomes. The extracellular domain of CD38 was fused to the Fc domain of a murine immunoglobulin G. These baitbody constructs were thoroughly characterized and applied in both serological microcolumn agglutination and automated solid‐phase red cell adherence assays. The CD38‐mFc baitbody successfully prevented agglutination reactions induced by three clinically relevant anti‐CD38 monoclonal antibodies—daratumumab, felzartamab and isatuximab—in spiked samples. This allowed the detection of alloantibodies of the Rhesus, Kell and Duffy blood groups without interference. The CD38‐mFc construct also demonstrated potential in a head‐to‐head comparison with commercial mitigation reagents, DaraEx and Grifols sCD38. Finally, the CD38‐mFc baitbody effectively neutralized daratumumab in patient samples, preventing false positive test outcomes.

## 1. Introduction

More than 200 therapeutic antibodies have received approval (EU EMA, US FDA, and other countries) for the treatment of hematologic and solid cancers, and many others are in phase III clinical studies (source: antibodysociety.org). These antibodies are directed against cell surface protein markers on cancer cells. One such monoclonal therapeutic antibody is daratumumab. In preclinical investigations, daratumumab exhibited significant cytotoxicity towards neoplastic cells through diverse pathways, such as complement‐dependent cytotoxicity and antibody‐dependent cellular cytotoxicity, inducting apoptosis in these cells [1].

Besides the therapeutic effects of daratumumab, it can interfere with the immunophenotyping of tumor cells [2]. However, a far more relevant problem is caused with the provision of compatible blood products in transfusion medicine [3]. Daratumumab targets CD38, an antigen which is highly expressed on the surface of multiple myeloma cells [4, 5], but can also be found on erythrocytes [6, 7]. Therefore, anti‐CD38 therapeutic antibodies such as daratumumab are causing interference in the sensitive indirect anti‐globulin test (IAT) [8]. The binding of daratumumab to CD38 causes crosslinking of red blood cells, this results in agglutination of the tested cells, a phenomenon known as panagglutination, leading to false positive results in the IAT. This (pan)agglutination caused by daratumumab, masks the detection of relevant anti‐erythrocyte alloantibodies, like those targeting the Rhesus, Kell, Duffy, Kidd, and MNS blood group systems. Unfortunately, this effect arises in patients who are undergoing treatment with daratumumab or another anti‐CD38 antibody. These patients frequently need blood transfusions while receiving treatment. But, because these antibodies are affecting the results of routine blood compatibility testing methods, it becomes challenging to detect the presence of relevant anti‐erythrocyte alloantibodies for the patient [9, 10]. Consequently, this interference hinders the ability to match and administer compatible donor blood for the necessary transfusions. There are several options mentioned in the literature for solving this daratumumab interference [5, 11] but they are all either cost‐ or time‐intensive or may interfere with other blood group antigens on erythrocytes. To tackle this issue, we developed a unique “baitbody” strategy involving a CD38‐Fc fusion protein. Notably, we have previously employed a comparable baitbody approach effectively to target distinct anti‐ *N*‐methyl‐*D*‐aspartate (NMDAR) receptor autoantibodies [12]. In this study, we adapted this approach to exclusively target therapeutic antibodies against CD38 within the blood samples obtained from patients and therefore, used it for the mitigation of anti‐CD38 antibody interference in serological tests, using both microcolumn agglutination assays and automated solid‐phase red cell adherence assays.

## 2. Material and Methods

### 2.1. Daratumumab Treated Patient Samples

Blood samples (serum and plasma) from patients treated with daratumumab were collected at the Institute for Clinical Transfusion Medicine Unit at Braunschweig Municipal Hospital. Every willing donor was provided with information about the project’s details and provided with written consent. The utilization of blood samples received ethical approval from the Ethik‐Kommission der Ärztekammer Niedersachsen (approval Bo/64/2021).

### 2.2. “Baitbody” Design and Production

For the construction of the CD38 baitbody, the extracellular domain (aa 43‐300, Uniprot ID P28907) was subcloned into the pCSE2.6‐mIgG2a‐Fc‐XP [13] and pCSE2.5‐His‐XP [14] vectors using *Nco*I/*Not*I (New England Biolabs, Frankfurt, Germany) for production. The CD38 baitbody, the extracellular domain of CD38 with a His‐Tag for purification and the NMDAR‐Fc baitbody, which served as a control fusion protein, were produced in mammalian Expi293F cells as described before [12]. Expi293F cells were maintained at 37°C, 110 rpm, and 5% CO2 in Gibco FreeStyle F17 expression medium (Thermo Fisher Scientific), augmented with 8 mM Glutamine and 0.1% Pluronic F68 (PAN Biotech). The transfection was executed when cell density reached approximately 2 × 10^6^ cells/mL, with a viability of approximately 85%–90%. For the assembly of DNA:PEI complexes, 1 μg DNA/mL of transfection volume and 5 μg of 40 kDa PEI (Polysciences) were initially separately diluted in 5% of the transfection volume using supplemented F17 medium. The DNA and PEI were then combined and incubated at approximately 25°C for around 25 min before introduction to the cells. Following a 48 h incubation, the culture volume was doubled by supplementing with HyClone SFM4Transfx‐293 medium (GE Healthcare), fortified with 8 mM Glutamine. Additionally, 10% of the final volume of HyClone Boost 6 supplement (GE Healthcare) was incorporated. Harvesting was performed 1 week post‐transfection through a 15 min centrifugation at 1500×*g*.

### 2.3. “Baitbody” Purification

Purification was executed following the methodology outlined in Steinke et al. [12], employing a 1‐mL column on Äkta go (Cytiva), Äkta Pure (Cytiva), or Profinia System (BIO‐RAD). All purification procedures were performed according to the manufacturer instructions.

### 2.4. SDS‐PAGE and Coomassie Staining

All generated baitbody proteins were subjected to analysis using sodium dodecyl sulfate‐polyacrylamide gel electrophoresis (SDS‐PAGE) coupled with Coomassie staining or Western blot. A 12% sodium dodecyl sulfate‐polyacrylamide gel was prepared as per the manufacturer’s instructions (BIO‐RAD). For assessment, 32 μL of the produced supernatant was combined with 8 μL of 5x Laemmli buffer containing ß‐mercaptoethanol and incubated at 95°C for 10 min. After a brief cooling period and 20 s centrifugation, 15 μL of each sample was loaded onto the 12% gel. Furthermore, 8 μL of the BIO‐RAD Precision Plus Protein All Blue Standard marker was included. The gel ran at 25 mA and 300 V in SDS running buffer (25 mM Tris; 192 mM Glycine; 0.1% SDS). Following the electrophoretic separation, the gel was stained overnight with Coomassie Brilliant Blue solution (0.05% Coomassie‐Brilliant Blue R250; 10% acetic acid; 25% isopropanol) or blotted onto PVDF membrane. The destaining of the Coomassie gel was performed on the following day for 2 h using 10% acetic acid and subsequently, the decolorized gels were documented using Bio‐Rad’s camera system (ChemiDoc MP).

### 2.5. Binding Enzyme‐Linked Immunosorbent Assay (ELISA)

A binding ELISA was conducted by immobilizing 200 ng of the CD38 baitbody onto 96‐well microtiter plates (High binding, Costar) in PBS with a pH of 7.4 overnight at 4°C. After immobilization, the wells were blocked with 350 μL of 2% M‐PBST (2% milk powder in PBS containing 0.05% Tween20). After blocking, the wells underwent three washes using H_2_O with 0.05% Tween20. Serial dilution of the anti‐CD38 antibody (daratumumab, isatuximab or felzartamab) was introduced, beginning at a 1:√10 dilution starting from 140 nM. Following a 1 h incubation and three subsequent washes with H_2_O containing 0.05% Tween20, binding was detected by employing goat‐anti‐human IgG(Fc)‐HRP (1:70000, A0170, Sigma). The binding event was visualized using a tetramethylbenzidine (TMB) substrate composed of TMB‐A (30 mM potassium citrate; 1% w/v citric acid, pH 4.1) and TMB‐B (10 mM TMB; 10% v/v acetone; 90% v/v ethanol; 80 mM H_2_O_2_) in a 20:1 ratio. The TMB reaction proceeded for 30 min, and the resulting absorbance was measured at 450 nm, with a reference wavelength of 620 nm, using an ELISA plate reader (Epoch, BioTek).

### 2.6. Antibody Screening With the Indirect Anti‐Globulin Test (IAT)

Unless mentioned otherwise, the IATs were carried out following the instructions provided by the manufacturer using the Bio‐Rad system. In simpler terms, 50 µL 0.8% test erythrocytes of a three‐cell panel (ID‐DiaCell I‐II‐III, Bio‐Rad) were incubated with 25 µL patient or donor serum or plasma sample. The mixture was incubated at a temperature of 37°C for 15 min in small cards containing a special gel called ID‐Cards LISS/Coombs. This gel has antibodies that can detect specific parts of the immune system (IgG and C3d). After 15 min, the cards were spun in an ID‐centrifuge for 10 min at 1030 rpm. The result, whether the mixture caused clumping (agglutination) of the substances, was assessed and documented by photography. The antigen phenotypes of the three‐cell panel (ID‐DiaCell I‐II‐III, Bio‐Rad) are listed in the Supporting Information (Table S1).

For the competition experiments, the therapeutic anti‐CD38 antibodies, daratumumab, isatuximab and felzartamab, the blood group alloantibodies, anti‐D, anti‐K and anti‐Fy^a^ from the Validation Panel (Werfen), and different mitigation reagents were added to donor plasma or serum samples prior to the IATs. The therapeutic anti‐CD38 antibodies were added in concentrations 100 nM, 500 nM or 1400 nM. For daratumumab 1400 nM is equivalent to 0.5 mg/mL, which is in the range of the expected peak serum concentration after therapy [15]. For the mitigation, the CD38‐mFc baitbody, the soluble extracellular domain of CD38 with an His‐Tag and the control N1‐N2B‐mFc baitbody were added to the serum or plasma in threefold excess or molar equivalent and incubated for 15 min at room temperature. The commercially available mitigation reagents Grifols sCD38 and DaraEx (Imusyn) were applied according to the manufacturer protocols.

For the processing of patient samples, CD38‐mFc was introduced into the samples at a concentration of 4160 nM or 5200 nM. To assess cross‐reactivity with the murine Fc‐domain, an equivalent concentration of the control baitbody, N1‐N2B‐mFc, was employed. Furthermore, the impact of therapeutic antibody dilution was investigated, utilizing a PBS volume control equivalent to the added baitbody volume.

### 2.7. Identification Panel With Capture‐R Ready‐ID on the NEO

Blood samples were taken from the donors with S‐Monovette 7.5 mL 92 × 15 mm, K3 EDTA tubes (Sarstedt). For each approach 2.5 mL blood was prepared in separate tubes. For the addition of the red blood cell alloantibodies (anti‐K, anti‐D and anti‐ Fy^a^), 100 µl of the respective validation panel samples was added. Daratumumab was added in a concentration of 500 nM. The baitbody reagent was added in a threefold molar excess to the daratumumab concentration. After the addition of the components, the samples were centrifuged for 3 min at 5000 rpm. The samples were given sample IDs with unique bar codes, and the automated system NEO (Werfen) was used. Each sample was tested with the Capture‐R Ready‐ID kit (LOT: ID466, expiration date: 2024‐05‐07). These tests are on a micro titer plate basis with each test consisting of 2× 8 well strips. Each well is coated with the reagent red blood cell stroma of 14 unique O single donors with defined antigen composition (Table S2) as well as a positive and negative control. Each well was filled with 50 µl of Low Ionic Strength Saline (LISS) and 25 µl of the sample. The strips were incubated for 20 min at 37°C and subsequently washed. The indicator group O cells coated with anti‐human IgG are added to each well and the plates were then centrifuged for 2 min at 530 rpm. Each well is photographed with the camera reader, and the photographs are analyzed, producing raw values between −2 and +100. The system presents the results as reaction (−, ?, +) as grade (−, ?, 1, 2, 3, 4) and reaction strength (0.00–99.9). After the completion of each test, the results were reviewed and compared to a visual assessment of the plates.

## 3. Results

### 3.1. CD38 Baitbody Construct Shows High Specificity to the Therapeutic Anti‐CD38 Antibodies Daratumumab, Isatuximab and Felzartamab

For the development of the baitbody protein, the extracellular domains of CD38 (amino acid 43–300) were fused to a murine IgG2a Fc domain (Figure 1A). With this protein design choice, a soluble bivalent CD38‐mFc fusion molecule was generated. The producibility yield of the CD38 baitbody construct in EXPI293 cells was 25 mg/L and the molecular size of the protein was at approximately 116 kDa, with each chain linked through disulfide bonds (Figure 1B). To evaluate the binding effectiveness of the CD38‐mFc baitbody to the therapeutic anti‐CD38 antibodies, an ELISA was conducted. The CD38‐Fc fusion was immobilized onto microtiter plates and the monoclonal therapeutic antibodies daratumumab, isatuximab and felzartamab were titrated. It was observed that the baitbody exhibited a strong binding interaction to all the three tested therapeutic anti‐CD38 antibodies (Figure 1C). The estimated affinity (EC50‐values) for daratumumab was approximately 0.12 nM, while for isatuximab and felzartamab it was around 0.18 nM and 0.32 nM. The ELISA procedure was also conducted in an inverted manner and showed similar results, referring to Supporting Information Figure S1 for details. This data indicates that the engineered CD38‐mFc baitbody effectively engages in a robust and target specific interaction with the therapeutic anti‐CD38 antibodies.

Figure 1An engineered CD38‐Fc‐fusion construct has a high affinity for therapeutic anti‐CD38 antibodies. (A) Illustration of a fusion construct of CD38 and a murine IgG Fc part with an illustration of the domains of the Fc fusion construct. (B) Results of SDS‐Page under reducing conditions with Coomassie staining of the single chains fusion construct at approximately 60 kDa (mFc = mouse Fc). (C) The relative absorbance (420–650 nm) correlating to the binding intensity was plotted against the IgG concentration in nM. The bound daratumumab, isatuximab and felzartamab to immobilized baitbody were detected with a goat anti‐human IgG‐ peroxidase (A0170). The N1‐N2B‐mFc baitbody was used as a control for the binding specificity. All steps were carried out in 2% MPBST at room temperature (20°C).

**Figure:**
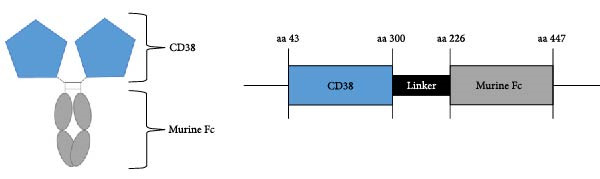
(A)

**Figure:**
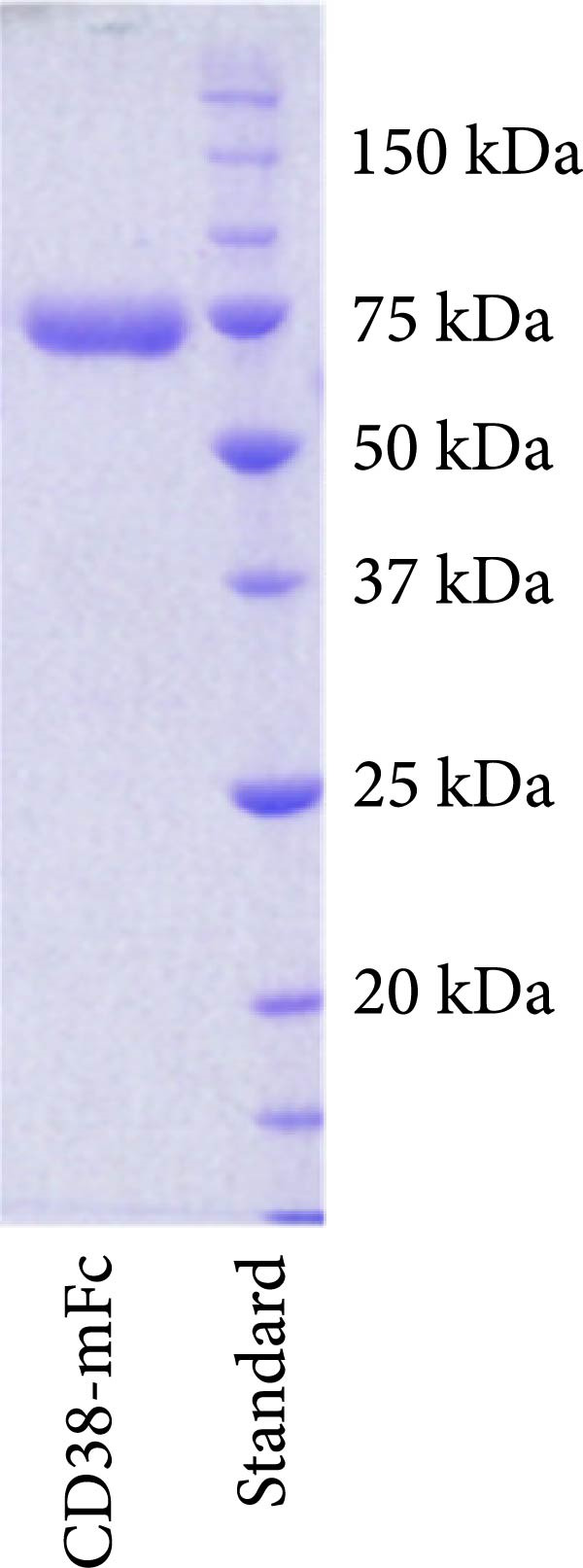
(B)

**Figure:**
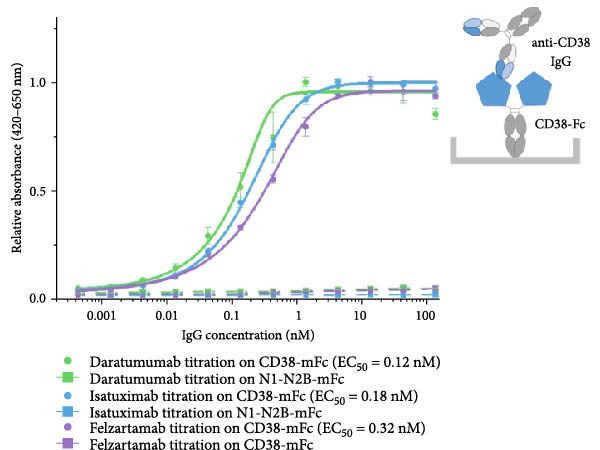
(C)

### 3.2. CD38 Baitbody Shows Excellent Inhibition of Daratumumab, Isatuximab and Felzartamab in Spiked Samples in the Indirect Antiglobulin Test

The efficacy of the baitbody construct CD38‐mFc in preventing false positives results in the indirect antiglobulin test (IAT) was evaluated using donor plasma spiked with daratumumab, isatuximab and felzartamab. While all three anti‐CD38 antibodies induced false positive IATs, the degree of agglutination reactions varied.

Daratumumab elicited the most robust panagglutination reactions, reaching up to 2+ with all three ID‐DiaCell types (ID‐DiaCell I‐II‐III, Bio‐Rad) at a concentration of 100 nM (Figure 2A). To assess mitigation capabilities, the baitbody was introduced at both a three‐fold molecular excess and a molar equivalent. The same comparative analysis was performed with soluble CD38‐His and N1‐N2B‐mFc (a control baitbody with an identical Fc‐domain [12]). Complete mitigation was achieved with the addition of CD38‐mFc in a three‐fold excess, whereas interference persisted at the molar equivalent. Soluble CD38‐His did not prevent agglutination at these ratios (Figure S2A). Isatuximab induced similarly robust agglutination reactions in the IAT of ID DiaCell type I like daratumumab, while reactions in the remaining cell types were minimal at 100 nM (Figure S2B). Neutralization tests were conducted at the same ratios and with the same fusion proteins. Despite the intensity of the reaction, both CD38‐mFc and CD38‐His prevented agglutination at a molar equivalent (Figure 2B). The control baitbody construct N1‐N2B‐mFc exhibited no discernible effects. Felzartamab showed a weaker reaction at the same concentration of 100 nM, resulting in macroscopically positive IATs for all three ID cell types (Figure S2C). Soluble CD38‐His mitigated interference at a three‐fold excess, while CD38‐mFc prevented agglutination at a 1:1 ratio (Figure 2C).

Figure 2Results of IATs using plasma spiked with daratumumab, isatuximab and felzartamab. (A) The indirect antiglobulin tests (IATs) were carried out with human plasma (A1) with the addition of 100 nM daratumumab (A2‐8). (B) Human plasma (B1) was spiked with 100 nM isatuximab (B2‐8). (C) Human plasma (C1) was also spiked with 100 nM felzartamab (C2‐8). The neutralization of daratumumab, isatuximab and felzartamab was tested with CD38mFc, CD38‐His and N1‐N2B‐mFc. CD38‐mFc baitbodies were added in 3:1 (A3, B3, C3) and 1:1 ratios (A4, B4, C4), CD38‐His in a 3:1 (A5, B5, C5) and 1:1 ratio (A6, B6, C6) as well as N1‐N2B‐mFc in 3:1 (A7, B7, C7) and 1:1 ratios (A8, B8, C8). The erythrocytes used were ID‐DiaCells I, cells II and cells III. The IAT images presented here were conducted using ID‐DiaCells I. Images for cells II and III can be found in the Supporting Information. After plasma and erythrocytes were mixed on ID card test columns, the cards were incubated for 15 min at 37°C and centrifuged for 10 min at 910 rpm. Afterwards the agglutination reaction was documented via photography.

**Figure:**
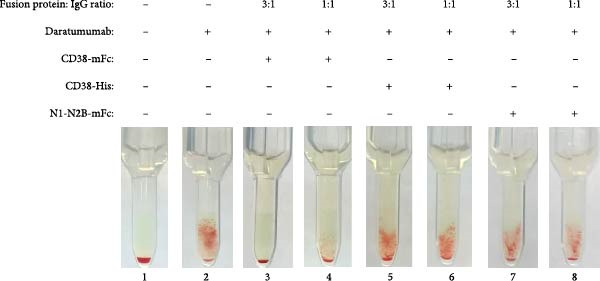
(A)

**Figure:**
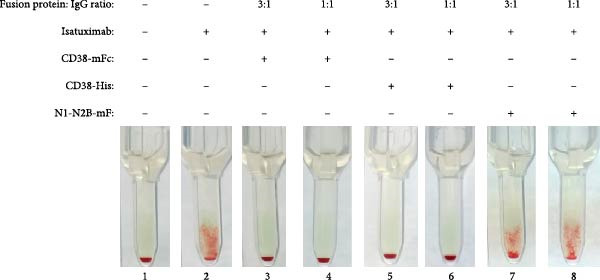
(B)

**Figure:**
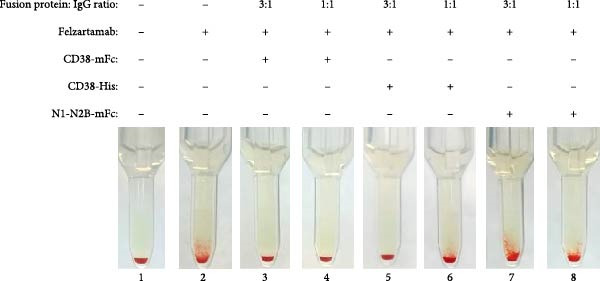
(C)

### 3.3. CD38 Baitbody Inhibition of Daratumumab Enables Identification of Irregular Alloantibodies

An essential prerequisite for any mitigation strategy is to avoid interference with antigens or alloantibodies central to blood group systems. To assess whether the addition of baitbodies hinders the detection of alloantibodies, antibody screening IATs were conducted using test serum containing alloantibodies against the D antigen of the Rhesus blood group system, K antigen of the Kell blood group system or Fy^a^ antigen of the Duffy blood group system. The presence of the antigen on the test erythrocytes and the corresponding alloantibodies in the serum induced agglutination, resulting in a specific pattern.

Daratumumab, at a concentration of 500 nM, induced the characteristic panagglutination, effectively masking the presence of alloantibodies. Given the successful complete mitigation observed with a three‐fold excess of CD38‐mFc, as detailed above, the same ratio was applied in this experiment. Introduction of the baitbody construct to the spiked serum prevented panagglutination, resulting in the same agglutination pattern for the anti‐D (Figure 3A), anti‐K (Figure 3B) and anti‐Fy^a^ (Figure 3C) alloantibodies as prior to the daratumumab addition.

Figure 3Results of antibody screening IATs using serum spiked with anti‐D, anti‐K or anti‐Fy^a^ alloantibodies and daratumumab. The indirect antiglobulin tests were carried out with test human serum with anti‐D (A), anti‐K (B) and anti‐Fy^a^ (C) alloantibodies and ID‐DiaCells I, cells II and cells III as test erythrocytes (1–3). ID‐DiaCell phenotypes are listed Table S1 in the Supporting Information. 500 nM daratumumab was added (4–6) and competition was carried out with CD38‐mFc in a 3:1 ratio (7–9). After plasma and erythrocytes were mixed on the ID card test columns, the cards were incubated for 15 min at 37°C and centrifuged for 10 min at 910 rpm. Afterwards the agglutination reaction was documented via photography.

**Figure:**
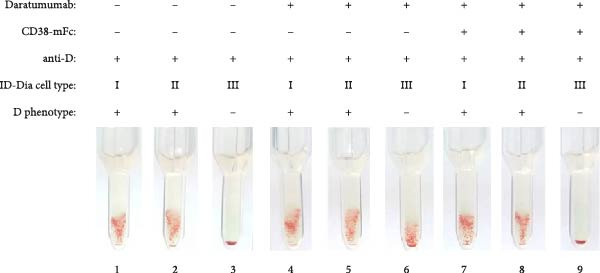
(A)

**Figure:**
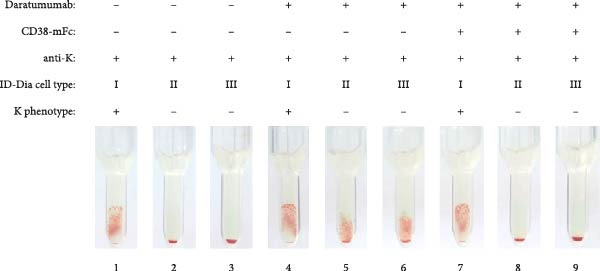
(B)

**Figure:**
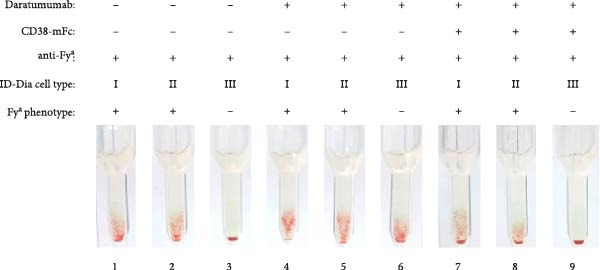
(C)

Following the antibody screening in the IAT format, the analysis was expanded to include an antibody identification 14 cell panel with the solid phase Capture‐R method on the automated NEO system (Werfen). The detection of antibodies was achieved in the solid phase method by the antibodies in the sample binding to the antigens on the coated cells and then being bound by anti‐human IgG coated on indicator cells. Through a centrifugation step, unbound indicator cells are pooled in the center of the well, while bound indicator cells remain spread out. The results of the antibody identification panel for the anti‐D (Figure S3), anti‐K (Figure S4), and anti‐Fy^a^ (Figure S5) samples without daratumumab showed clear reaction patterns which corresponded with the phenotypes of the panel cells. The addition of daratumumab, at a concentration of 500 nM, caused varying reaction levels for each of the panel cells and reaction was graded one for several cells (Figure 4). This made the antibody identification no longer conclusive. The interference caused by daratumumab was mitigated through the addition of a threefold molar excess of CD38‐mFc baitbody, enabling the antibody identification.

Figure 4Results of antibody identification Capture‐R® Ready‐ID® panel using serum spiked with anti‐K or anti‐Fy^a^ alloantibodies and daratumumab. The antibody identification panel was carried out with a sample of test human serum with anti‐K (A) or anti‐Fy^a^ (B) alloantibodies on the NEO system. 500 nM daratumumab was added, and competition was carried out with CD38‐mFc in a 3:1 molar ratio. The Capture‐R Ready‐ID panel consisted of 14 unique reagent red blood cell stroma coated on individual wells as well as a positive and negative control. Of this panel the results for cells 2, 5, 6, 8, and 13 and the respective K‐ or Fy^a^‐phenotypes are depicted. The complete 14 cell panel results (Figures S4 and S5) and phenotypes (Table S2) are shown in the Supporting Information. Each well was filled with 50 µl of Low Ionic Strength Saline (LISS) and 25 µl of the sample. The strips were incubated for 20 min at 37°C and subsequently washed. The indicator group O cells coated with anti‐human IgG are added to each well and centrifuged for 2 min at 530 rpm. After the centrifugation, unbound indicator cells are pooled in the center of the well, while bound indicator cells remain spread out. Each well was documented and analyzed by the NEO system. The reaction strengths were evaluated and presented by the system as a grade (0, +, ++, +++, ++++). After the completion of each test and visual assessment of the plates, the final strength, grade and image of the wells were documented.

**Figure:**
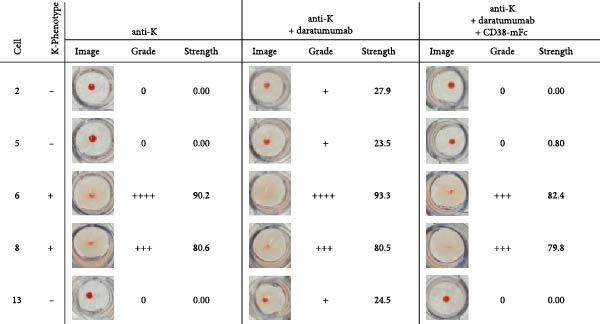
(A)

**Figure:**
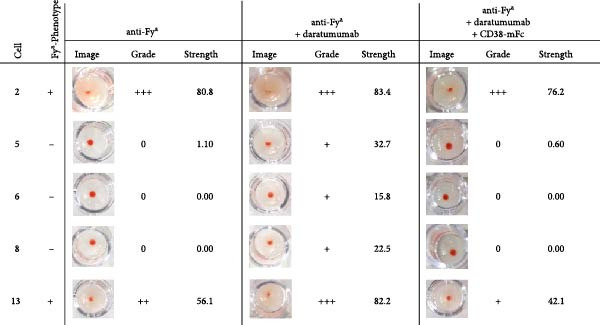
(B)

### 3.4. CD38 Baitbody Shows Improved Applicability in Comparison to Commercially Available Reagents

Different mitigation strategies have been proposed to address the interference caused by anti‐CD38 antibodies and have recently resulted in commercially available reagents. The first such reagent was DaraEx. DaraEx utilizes the Fab and Fab2 fragments of daratumumab to block CD38 on the surface of red blood cells [5]. The reagent from Grifols involves using the soluble or fusion protein with the CD38 receptor to neutralize anti‐CD38 antibodies [16].

**Figure 5:**
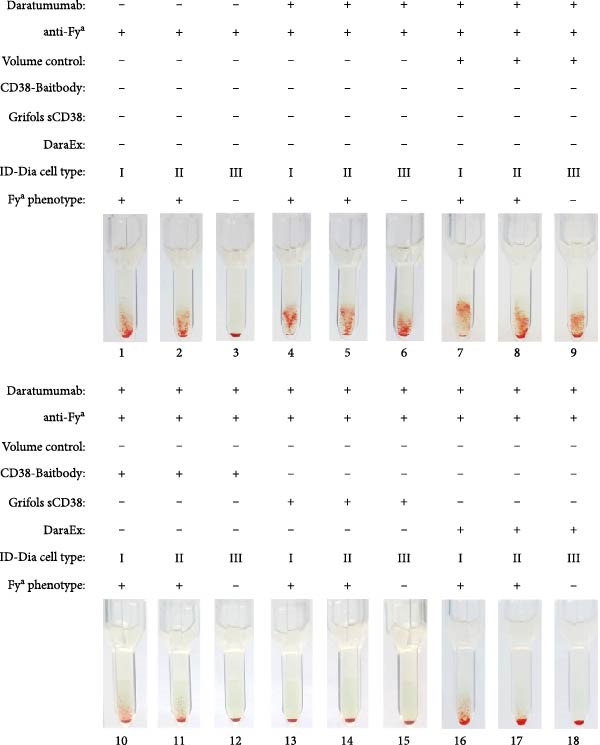
Results of antibody screening IATs using serum spiked with anti‐Fya alloantibodies and Daratumumab comparing different mitigation methods. The indirect antiglobulin tests were carried out with test human plasma with anti‐Fya alloantibodies and ID Dia cells I, cells II and cells III as test erythrocytes. ID Dia cell phenotypes are listed in Table S1. The plasma was spiked with 0.5 mg/mL Daratumumab, and competition was carried out with CD38‐mFc in a 3:1 molar ratio and Grifols sCD38 according to the manufacturer protocol. For the mitigation with DaraEx, the reagent was added to the erythrocytes according to the manufacturer protocol. After plasma and erythrocytes were mixed on the ID card test columns, the cards were incubated for 15 min at 37°C and centrifuged for 10 min at 910 rpm. Afterwards the agglutination reaction was documented via photography. After plasma and erythrocytes were mixed on ID card test columns, the cards were incubated for 15 min at 37°C and centrifuged for 10 min at 910 rpm. Finally, the agglutination reaction was documented via photography.

The effectiveness of the different commercial strategies in comparison to the CD38‐baitbody was tested in IATs with donor plasma supplemented with daratumumab at a concentration of 1400 nM in addition to anti‐Fy^a^ alloantibodies (Figure 5). While the three tested reagents all prevented the agglutination caused by daratumumab, the detection of the Fy^a^ alloantibodies was only possible after the mitigation with the CD38‐Baitbody and Dara‐Ex. After the application of the sCD38 reagent from Grifols, no agglutination reactions were observable for the positive Fy^a^ phenotyped ID‐DiaCells I and II (ID‐DiaCell I‐II‐III, Bio‐Rad).

### 3.5. CD38 Baitbody Protects Daratumumab Treated Patients From Agglutination Effects in IAT

**Figure 6:**
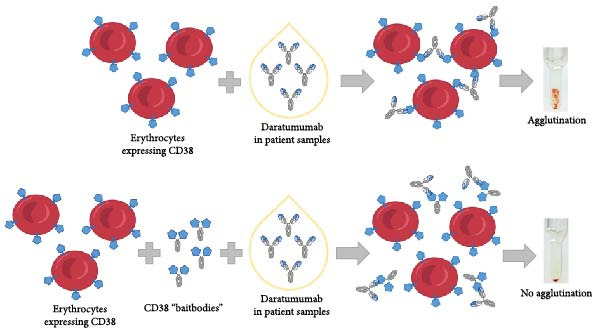
Illustration of the interference caused by daratumumab in patient samples during pre‐transfusion testing and mitigation strategy utilizing CD38 “baitbodies”. CD38 is expressed on the surface of erythrocytes and can be bound by anti‐CD38 therapeutic antibodies (daratumumab, isatuximab and felzartamab), resulting in false positive agglutination reactions and interfering with the screening for and identification of transfusion relevant alloantibodies. As a mitigation strategy, CD38‐Fc‐fusion constructs are added to bind the therapeutic antibodies and prevent the false positive agglutination reactions.

Following the assessment of baitbody competition with daratumumab in spiked plasma and serum samples, the efficacy of CD38‐mFc was examined in patient‐derived samples (Figure 6). These serum specimens were obtained from individuals undergoing treatment for multiple myeloma with daratumumab.

**Figure 7:**
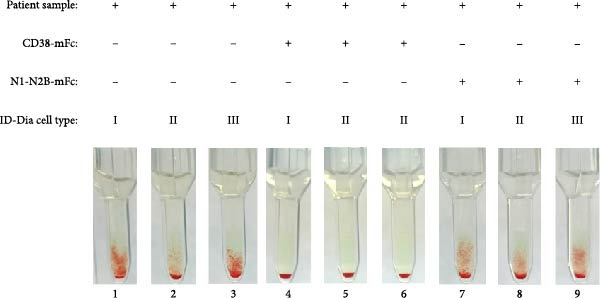
Results of indirect antiglobulin tests using patient samples with daratumumab. The indirect antiglobulin tests were carried out with serum samples from patient 1, who was treated with daratumumab (1–3). The erythrocytes used were ID Dia cells I, cells II and cells III. 4160 nM CD38‐mFc (4–6) or N1‐N2B‐mFc (7–9) was added to the serum. After serum and erythrocytes were mixed on ID card test columns, the cards were incubated for 15 min at 37°C and centrifuged for 10 min at 910 rpm. Afterwards the agglutination reaction was documented via photography. Patient 1 is shown as an example and the images for the remaining patients can be found in Supporting Information.

The IAT was performed with nine daratumumab patient samples. The results for patient 1 are given in Figure 7. Here, complete prevention of the agglutination reaction following the initial addition of CD38‐mFc, while the introduction of the control baitbody and dilution through PBS had no discernible effect on the agglutination level. The baitbody competition with CD38‐mFc for all nine patient samples is given in Table 1. The agglutination levels induced by daratumumab in patient samples exhibited relative consistency, estimated at +1 or +2 (Figure S6). Complete mitigation of agglutination was observed at the initial baitbody concentration of 4160 nM in patient samples 1, 2, and 5–9. However, at the same initial concentration, the IATs for patient 3 remained macroscopically positive for ID cells I and III. The IAT for ID cell II was negative. Subsequently, an increase in CD38‐mFc concentration to 5200 nM eliminated agglutination in patient 3. Similarly, patient 4 displayed agglutination at a +1 level for ID cells I and II, while the IAT for ID cell III exhibited no macroscopic reaction. Elevating the CD38‐mFc concentration resulted in decreased agglutination, with the IAT for ID cell I showing no macroscopic agglutination. None of the patients exhibited known RBC alloantibodies.

**Table 1:** Results of indirect antiglobulin tests using nine patient samples with daratumumab.

Patient	CD38‐mFc concentration (nM)	Agglutination (0 to ++++)		
		Cell I	Cell II	Cell III
1	‐ 4160 nM	+ 0	+ 0	+ 0
2	‐ 4160 nM	++ 0	++ 0	++ 0
3	‐	+	+	+
	4160 nM	±	0	±
	5200 nM	0	0	0
4	‐	++	++	+
	4160 nM	+	+	0
	5200 nM	0	±	0
5	‐	+	+	+
	4160 nM	0	0	0
6	‐	++	+	++
	4160 nM	0	0	0
7	‐	++	++	+
	4160 nM	0	0	0
8	‐	+	+	++
	4160 nM	0	0	0
9	‐	++	+	+
	4160 nM	0	0	0

*Note*: The indirect antiglobulin tests were carried out with serum samples from patients 1–9, who were treated with daratumumab. The erythrocytes used were ID Dia cells I, cells II, and cells III. The IATs were first performed with the patient serum samples and the level of agglutination reactions was documented and graded. For the mitigation, 4160 nM CD38‐mFc was added to the serum. If the agglutination persisted, the IAT was repeated with 5200 nM CD38‐mFc. After plasma and erythrocytes were mixed on ID card test columns, the cards were incubated for 15 min at 37°C and centrifuged for 10 min at 910 rpm. Afterwards the agglutination reaction was documented via photography and graded (0 to ++++). The images for the remaining patients can be found in Supporting Information.

## 4. Discussion

Anti‐CD38 therapeutic antibodies have been fundamental in advancing the treatment of multiple myeloma [17, 18], while gaining relevance through an increasing number of off‐label uses and clinical trials for the treatment of further plasma cell disorders and hematologic malignancies [19–22] as well as treatment of kidney disorders [23–27], autoimmune disorders [27, 28], antibody‐mediated rejection after organ transplantation [29–31] and desensitization prior to transplantation [32, 33]. Even without these new applications, anti‐CD38 therapeutic antibodies are already notorious in transfusion laboratories worldwide due to interfering with routine antibody screening and crossmatching workflows [3]. The current standard to mitigate this interference is the use of dithiothreitol (DTT) to reduce disulfide bonds and thus denature CD38 on the surface of erythrocytes. Notably, several blood group systems are also disrupted by DTT, including Kell, Lutheran, YT, JMH, LW, Cromer, Indian, Dombrock, and Knops [34]. This method additionally involves several washing and incubation steps, prolonging the processing time of these samples and occurring additional costs [35].

In this study, Fc‐fusion proteins, denoted as baitbodies, were developed to prevent the interference caused by anti‐CD38 therapeutic antibodies and address the disadvantages of the current DTT‐methods. Fc‐fusion proteins offer versatile applications due to the diverse fusion partners they can accommodate [12, 36, 37]. While designing the baitbody, the selection of the murine Fc domain, as opposed to the human IgG1, was a deliberate choice to prevent potential interactions with anti‐human IgG antibodies within the hematological antibody screening and identification assays. Since therapeutic anti‐CD38 antibodies target the extracellular domain of CD38, the transmembrane and intracellular domains were omitted in the development of the Fc fusion baitbody protein. Nevertheless, incorporating the full extracellular domain of CD38 was vital to encompass all the possible epitopes. This epitope coverage was demonstrated in the functional validation of CD38‐mFc, with the construct binding to daratumumab, isatuximab, and felzartamab and preventing the agglutination reactions caused by these antibodies in IATs with spiked samples. Daratumumab and isatuximab have been shown to bind distinct CD38 epitopes and further anti‐CD38 antibodies in development may recognize new epitopes [1, 38, 39]. There are first indications that the epitope of felzartamab may be partially resistant to the treatment with DTT [40], underlining the need for alternatives to the DTT method.

The mitigation of false positive reactions caused by anti‐CD38 antibodies was also achieved in automated solid‐phase red cell adherence assays, demonstrating compatibly of CD38‐mFc with different routine cross matching techniques. In both the microcolumn and solid‐phase assays, the application of CD38‐mFc was simple and time‐effective, as the reagent only had to be added to the plasma, serum or blood sample prior to the tests. This did not require any additional time‐consuming washing steps such as DTT treatment of test erythrocytes [41] or adsorption rounds on CD38‐expressing cells [42]. The simplicity of the method and applicability in high‐throughput assays could reduce the time required to process samples of patients treated with anti‐CD38 therapeutics. While the cost of protein‐based mitigation methods has been considered the largest drawback compared to the DTT strategy [11], CD38‐mFc has the potential to reduce labor costs and handling time. Additionally, advances in the field of protein production and purification have increased efficiency and reduced protein production costs [43, 44].

The baitbody construct was also observed to be more effective compared to the soluble CD38‐His variant, allowing full mitigation at a lower molar ratio. This was advantageous at high therapeutic antibody concentrations, while using concentrated protein regents to avoid significant dilution effects. Peak daratumumab serum concentration of approximtely 1 mg/mL and 0.6–0.7 mg/mL for isatuximab were indicated by pharmacokinetic analysis [15, 45]. While felzartamab is still in development and pharmacokinetic data is not yet available, current clinical trials utilize the same recommended dose as daratumumab which would likely result in a similar range of peak serum concentration [29]. In this study, neutralization of daratumumab was tested up to 1400 nM which is equivalent to 0.5 mg/mL as well as in patient samples, showing that the technical requirements to mitigate the interference at high serum concentrations were applicable.

Neutralization of anti‐CD38 antibodies by the baitbody enabled the detection of the clinically relevant anti‐D, anti‐K and anti‐Fy^a^ alloantibodies in spiked serum. The detection of the anti‐K alloantibodies addressed the limitations of DTT‐based mitigation strategy [34]. These antigens were chosen to represent examples of different transfusion relevant blood groups, but a full evaluation with alloantibodies from a wide range of blood groups would still be necessary for IVD approval, to determine if any disruption could occur when using the CD38‐mFc reagent. This study also compared CD38‐mFc to two commercial mitigation reagents Dara‐Ex and Grifols sCD38. DaraEx needed to be added to each individual test tube containing different donor or identification panel erythrocytes prior to the mixture with the serum or plasma samples. In contrast the CD38‐mFc baitbody and the Grifols sCD38 reagent are added directly to the serum or plasma sample and the samples subsequently distributed to the individual tests. In the head‐to‐head comparison of the CD38‐mFc baitbody to the commercial reagent from Grifols, anti‐Fy^a^ alloantibodies reacted negatively after the use of the Grifols product. The mechanism of the Grifols sCD38 causing false negatives for antibodies against the Duffy A antigen is not yet understood [16]. A resulting drawback of the Grifols sCD38 is the requirement of Duffy A negative blood products. Despite the similarities in mitigation approach, the CD38‐mFc baitbody did not interfere with the detection of anti‐Fy^a^ alloantibodies.

Application of CD38‐mFc in patient samples treated with daratumumab demonstrated successful mitigation in the majority of cases, with variations possibly attributable to differing ID‐DiaCell sensitivities or alloantibody reactions. Despite these variations, CD38‐mFc baitbody showcased its potential as a tool for mitigating interference caused by anti‐CD38 therapeutic antibodies in serological tests.

## 5. Conclusions

Wider application of anti‐CD38 therapeutic antibodies and the ongoing development of new anti‐CD38 antibodies will add additional workload to the transfusion safety infrastructure. The engineered CD38‐mFc baitbody demonstrates the ability to prevent interference from anti‐CD38 therapeutic antibodies and secure the detection of clinically relevant alloantibodies. The baitbody approach provides advantages over existing mitigation strategies, with potential to address interferences caused by other therapeutic antibodies in diagnostic infrastructure. As therapeutic antibodies continue to gain prominence, a diverse toolkit of mitigation methods, including baitbodies, become essential to address interference in clinical laboratory tests.

## Conflicts of Interest

The authors declare a conflict of interest. Dianne Celine Gnann, Stephan Steinke, Hendrikus S. P. Garritsen, and Michael Hust are inventors on a patent application on CD38 baitbodies. The authors were employed at the Technische Universität Braunschweig or Städtische Klinikum Braunschweig gGmbH during this study.

## Author Contributions

Dianne Celine Gnann and Stephan Steinke contributed equally to this work. Hendrikus S. P. Garritsen and Michael Hust share senior authorship.

## Funding

Open Access funding enabled and organized by Projekt DEAL.

## Supporting Information

Additional supporting information can be found online in the Supporting Information section.

## Supporting information


**Supporting Information** Additional information can be found in the Supporting Information, which encompasses additional ELISA data, the tables listing the antigen lists of the ID‐erythrocyte panels and photographs of the complete results of the IAT (indirect antiglobulin test) panels and solid phase Capture‐R panels carried out in this study.

## Data Availability

The data that support the findings of this study are available from the corresponding author upon reasonable request.
