# Supplementary material for: Resolving the Interference of Anti‐CD38 Antibodies on Blood Compatibility Assays Using CD38 “Baitbodies” Approach
Source: J Immunol Res. 2026 Jan 4;2026:7343647. doi: 10.1155/jimr/7343647 (PMC12765988; doi:10.1155/jimr/7343647)
Supplement: Supplementary file 1 — Supporting Information Additional information can be found in the Supporting Information, which encompasses additional ELISA data, the tables listing the antigen lists of the ID‐erythrocyte panels and photographs of the complete results of the IAT (indirect antiglobulin test) panels and solid phase Capture‐R panels carried out in this study. [file JIMR-2026-7343647-s001.docx]

**Supplementary Material:**

**SupFigure 1: An engineered CD38-Fc-fusion construct has a high affinity for therapeutic anti-CD38 antibodies**

The relative absorbance (420-650nm) correlating to the binding intensity was plotted against the CD38-mFc concentration in nM. The bound CD38-mFc baitbody to immobilized Daratumumab, Isatuximab and Felzartamab were detected with a goat anti-mouse IgG-peroxidase (A0168). The human IgG 003-102-hIgG was used as a control for the binding specificity. All steps were carried out in 2% MPBST at room temperature (20°C).

**SupFigure 2: Results of IATs using plasma spiked with Daratumumab, Isatuximab and Felzartamab.**

A: The indirect antiglobulin tests (IATs) were carried out with human plasma with the addition of 100 nM Daratumumab. B: Human plasma was spiked with 100 nM Isatuximab. C: Human plasma was also spiked with 100 nM Felzartamab. The neutralization of Daratumumab, Isatuximab and Felzartamab was tested with CD38mFc, CD38-His and N1-N2B-mFc. CD38-mFc baitbodies were added in 3:1 and 1:1 ratios, CD38-His in a 3:1 and 1:1 ratio as well as N1-N2B-mFc in 3:1 and 1:1 ratios. The erythrocytes used were ID-DiaCells I, cells II and cells III. After plasma and erythrocytes were mixed on ID card test columns, the cards were incubated for 15 min at 37°C and centrifuged for 10 min at 910 rpm. Afterwards the agglutination reaction was documented via photography.

SupTable 1: Biorad ID-DiaCell I-II-III antigen list (Set IP-IIP-IIIP: 45194.62.x, expires: 2024/04/01), nt: not tested

SupTable 2: CAPTURE-R Ready-ID antigen list (LOT NO: ID466, expires: 2024/05/07)

**SupFigure 3: Results of antibody identification Capture-R® Ready-ID® panel using serum with spiked anti-D alloantibodies and Daratumumab.**

The antibody identification panel was carried out with a sample of test human serum with anti-D alloantibodies on the NEO**®** system. 500 nM Daratumumab was added and competition was carried out with CD38-mFc in a 3:1 molar ratio. The Capture-R® Ready-ID® panel consisted of 14 unique reagent red blood cell stroma coated on individual wells as well as a positive and negative control. The complete 14 cell panel phenotypes are shown in the supplementary material (SupTab. 2). Each well was filled with 50 µl of Low Ionic Strength Saline (LISS) and 25 µl of the sample. The strips were incubated for 20 minutes at 37°C and subsequently washed. The indicator group O cells coated with anti-human IgG are added to each well and centrifuged for 2 min at 530 rpm. Each well was documented and analyzed by the NEO**®** system. The reaction strengths were evaluated and presented by the system as a grade (0, +, ++, +++, ++++). After the completion of each test and visual assessment of the plates, the final strength, grade and image of the well was documented.

**SupFigure 4: Results of antibody identification Capture-R® Ready-ID® panel using serum with spiked anti-K alloantibodies and Daratumumab.**

The antibody identification panel was carried out with a sample of test human serum with anti-K alloantibodies on the NEO**®** system. 500 nM Daratumumab was added and competition was carried out with CD38-mFc in a 3:1 molar ratio. The Capture-R® Ready-ID® panel consisted of 14 unique reagent red blood cell stroma coated on individual wells as well as a positive and negative control. The complete 14 cell panel phenotypes are shown in the supplementary material (SupTab. 2). Each well was filled with 50 µl of Low Ionic Strength Saline (LISS) and 25 µl of the sample. The strips were incubated for 20 minutes at 37°C and subsequently washed. The indicator group O cells coated with anti-human IgG are added to each well and centrifuged for 2 min at 530 rpm. Each well was documented and analyzed by the NEO**®** system. The reaction strengths were evaluated and presented by the system as a grade (0, +, ++, +++, ++++). After the completion of each test and visual assessment of the plates, the final strength, grade and image of the well was documented.

**SupFigure 5: Results of antibody identification Capture-R® Ready-ID® panel using serum spiked anti-F_y_^a^ alloantibodies and Daratumumab.**

The antibody identification panel was carried out with a sample of test human serum with anti-Fy^a^ alloantibodies on the NEO**®** system. 500 nM Daratumumab was added and competition was carried out with CD38-mFc in a 3:1 molar ratio. The Capture-R® Ready-ID® panel consisted of 14 unique reagent red blood cell stroma coated on individual wells as well as a positive and negative control. The complete 14 cell panel phenotypes are shown in the supplementary material (SupTab. 2). Each well was filled with 50 µl of Low Ionic Strength Saline (LISS) and 25 µl of the sample. The strips were incubated for 20 minutes at 37°C and subsequently washed. The indicator group O cells coated with anti-human IgG are added to each well and centrifuged for 2 min at 530 rpm. Each well was documented and analyzed by the NEO**®** system. The reaction strengths were evaluated and presented by the system as a grade (0, +, ++, +++, ++++). After the completion of each test and visual assessment of the plates, the final strength, grade and image of the well was documented.

**SupFigure 6: Results of indirect antiglobulin tests using patient samples with Daratumumab.**

The indirect antiglobulin tests were carried out with serum samples from patient 1-9, who were treated with Daratumumab. The erythrocytes used were ID Dia cells I, cells II and cells III. CD38-mFc was added in a concentration of 4160 nM or 5200 nM to the serum samples. The control baitbody N1-N2B-mFc was added in the same concentration. For the dilution control, the same volume of PBS was added to the patient samples prior to the tests. After serum and erythrocytes were mixed on ID card test columns, the cards were incubated for 15 min at 37°C and centrifuged for 10 min at 910 rpm. Afterwards the agglutination reaction was documented via photography.
